# Prevalence and correlates of reproductive coercion across ten sites: commonalities and divergence

**DOI:** 10.1186/s12978-023-01568-1

**Published:** 2023-01-27

**Authors:** Shannon N. Wood, Haley L. Thomas, Georges Guiella, Fiacre Bazié, Rosine Mosso, Raimi Fassassi, Pierre Z. Akilimali, Mary Thiongo, Peter Gichangi, Sani Oumarou, Funmilola M. OlaOlorun, Elizabeth Omoluabi, Anoop Khanna, Simon Peter Sebina Kibira, Fredrick Makumbi, Michele R. Decker

**Affiliations:** 1 Department of Population, Family and Reproductive Health, Johns Hopkins Bloomberg School of Public Health, 615 N Wolfe St, E4009, Baltimore, MD 21205 USA; 2 Department of Population, Family and Reproductive Health, Bill & Melinda Gates Institute for Population and Reproductive Health, Johns Hopkins Bloomberg School of Public Health, Baltimore, USA; 3 Institut Supérieur des Sciences de la Population (ISSP/University of Ouagadougou), Ouagadougou, Burkina Faso; 4 Ecole Nationale Superieure de Statistique et Appliquee d’Abidjan (ENSEA), Abidjan, Côte d’Ivoire; 5 Kinshasa School of Public Health, Kinshasa, Democratic Republic of Congo; 6 International Centre for Reproductive Health-Kenya, Nairobi, Kenya; 7 Technical University of Mombasa, Mombasa, Kenya; 8 Department of Public Health and Primary Care, Faculty of Medicine and Health Sciences, Ghent University, Ghent, Belgium; 9 Institut National de la Statistique du Niger, Niamey, Niger; 10 College of Medicine, University of Ibadan, Ibadan, Nigeria; 11 University of the Western Cape, Cape Town, South Africa; 12 Indian Institute of Health Management Research, Sanganer, Jaipur, India; 13 Makerere University School of Public Health, Kampala, Uganda; 14 Johns Hopkins School of Nursing, Baltimore, USA

**Keywords:** Reproductive coercion, Violence, Contraception, National data

## Abstract

**Background:**

Reproductive coercion (RC) is a type of abuse where a partner asserts control over a woman’s reproductive health trajectories. Recent research emphasizes that RC experiences may differ within and across low- and middle-income countries (LMICs), as compared to higher income contexts, given social pressures surrounding childbearing. To date, nationally representative surveys have lacked comprehensive measures for RC, leading to gaps in understanding its prevalence and risk factors. Across eight LMICs (10 sites), we aimed to (1) validate the RC Scale; (2) calculate prevalence of RC and specific behaviors; and (3) assess correlates of RC.

**Methods:**

This analysis leverages cross-sectional Performance Monitoring for Action (PMA) data collected from November 2020 to May 2022. Analyses were limited to women in need of contraception (Burkina Faso n = 2767; Côte d'Ivoire n = 1561; Kongo Central, Democratic Republic of Congo (DRC) n = 830; Kinshasa, DRC n = 846; Kenya n = 4588; Kano, Nigeria n = 535; Lagos, Nigeria n = 612; Niger n = 1525; Rajasthan, India n = 3017; Uganda n = 2020). Past-year RC was assessed via five items adapted from the original RC Scale and previously tested in LMICs. Confirmatory factor analysis examined fit statistics by site. Per-item and overall prevalence were calculated. Site-specific bivariate and multivariable logistic regression examined RC correlates across the socioecological framework.

**Results:**

Confirmatory factor analysis confirmed goodness of fit across all sites, with moderate internal consistency (alpha range: 0.66 Cote d’Ivoire–0.89 Kinshasa, DRC/Lagos, Nigeria). Past-year reported prevalence of RC was highest in Kongo Central, DRC (20.3%) and lowest in Niger (3.1%). Prevalence of individual items varied substantially by geography. Polygyny was the most common RC risk factor across six sites (adjusted odds ratio (aOR) range: 1.59–10.76). Increased partner education levels were protective in Kenya and Kano, Nigeria (aOR range: 0.23–0.67). Other assessed correlates differed by site.

**Conclusions:**

Understanding RC prevalence and behaviors is central to providing woman-centered reproductive care. RC was most strongly correlated with factors at the partner dyad level; future research is needed to unpack the relative contributions of relationship power dynamics versus cultural norms surrounding childbearing. Family planning services must recognize and respond to women’s immediate needs to ensure RC does not alter reproductive trajectories, including vulnerability to unintended pregnancy.

## Plain Language Summary

Reproductive coercion (RC) is a type of abuse where a partner asserts control over a woman’s reproductive health trajectories. While RC was conceptualized in the United States, recent research highlights that it may be prominent in other geographies, including sub-Saharan Africa. Existing national surveillance programs, including the Demographic and Health Surveys, have included a single item on RC beginning in 2018. Given the phased approach to Demographic and Health Survey roll-out, no studies have examined this single item across diverse contexts. Further, this single item may miss the range of abusive experiences women face when seeking to manage their fertility. Using annual national cross-sections in 10 diverse contexts (eight countries), we sought to: (1) validate a comprehensive RC measure; (2) calculate prevalence of RC and specific behaviors; (3) understand risk factors for RC across contexts. We found that the comprehensive RC measure performed well across sites. Prevalence of past-year RC was highest in the Kongo Central region of the Democratic Republic of Congo (20.3%) and lowest in Niger (3.1%). Polygynous marriage was associated with increased risk of RC across six sites, whereas increased partner education levels were protective against RC in two sites. Understanding the prevalence of RC within a given context and range of specific abusive behaviors, as well as risk profiles, can help alert local service providers to women’s needs. A thorough understanding of commonalities and divergence of RC experiences and drivers across sites can help inform prevention and response programming to address RC and its health effects.

## Introduction

Reproductive coercion (RC) is a type of abuse where a partner asserts control over reproductive health trajectories—through interfering with pregnancy planning or a pregnancy outcome—despite the other partner’s pregnancy intentions [1–3]. The term “RC” was coined by Miller et al. based on facility-based interviews with adolescents in the United States [4], corroborated in adult populations [2, 5], and later attributed to behaviors indicative of reproductive abuse globally [6–12]. While this terminology was adopted based on findings from the United States, literature in low- and middle-income countries (LMICs), particularly in sub-Saharan Africa and South Asia, has long described the fertility pressures that women face and emergent tensions should fertility desires within a couple conflict [13, 14]. A more recent emergent research base highlights that RC is prominent in LMICs, but existing studies are limited by sample size [6, 8] and generalizability (i.e., among IPV survivors or married adolescents) [9, 15]. Despite heterogeneity in contexts, study populations, and measures, the evidence is clear that RC incurs considerable harm to women’s health via increasing risk for unintended pregnancy [1, 5, 11, 16].

To date, the most comprehensive measurement for RC is the RC Scale, developed in the United States [17, 18]. While the original RC Scale is not inclusive of all RC behaviors, specifically coercive control of pregnancy outcomes [3], it captures a range of pregnancy promoting behaviors with specified coercive intent. In sub-Saharan Africa, where condoms are more widely used for HIV prevention than pregnancy prevention, a modified version of this scale focused on pregnancy coercion has been recommended [9, 15].

Despite these measurement innovations, RC measurement at the national level remains sub-optimal. In 2018, the Demographic and Health Surveys (DHS) began including a single item on RC within their Phase 8 Model Women’s Questionnaires [“Has your (husband/partner) or any other family member ever tried to force or pressure you to become pregnant when you did not want to become pregnant?”] [19]. Given the phased approach to DHS roll-out, countries collect data at different timepoints, limiting comparability; further, to date, few countries have released public data on RC, limiting potential for cross-site analyses. Moreover, this single item may miss the range of abusive behaviors women face when seeking to control their fertility. In line with best practices for violence-related research, multi-item behavioral assessments may be preferable [20].

Moreover, understanding factors that may put women at risk for RC is critical for prevention and response, however, research on correlates of RC (i.e., risk factors) is limited given the dearth of quantitative data specific to RC in LMICs. One study, in Cote d’Ivoire, focused on RC and in-law abuse and found that in-law perpetrated RC was associated with ethnicity and marriage [8]. More recent research among intimate partner violence (IPV) survivors in Nairobi, Kenya found significant positive correlations between past 3-month RC and not wanting their last child at all, partner’s concurrent partnership, and inconvenient contraceptive method use; the same study found that increased couple communication was protective against RC [21]. Withstanding these few studies among specific sub-populations, large gaps remain in understanding who is most vulnerable to this unique form of abuse, as well as who may be protected. Further, insight is needed into how risk factors differ across cultural, geographic, and political contexts.

Given the lack of national data on RC, across ten LMIC sites (eight countries), this study aimed to: (1) validate the RC Scale; (2) calculate the prevalence of RC and specific behaviors; (3) assess correlates of RC in order to understand commonalities and divergence of risk and protective factors across contexts.

## Methods

### Overview of Performance Monitoring for Action

The present analyses use data from Performance Monitoring for Action (PMA), a research platform that administers annual panel and cross-sectional surveys at the household, female, and facility levels in eight countries in sub-Saharan Africa and Asia. PMA uses a multi-stage cluster design with probability proportional to size sampling of enumeration areas to obtain nationally or regionally representative estimates. Surveys are administered by trained resident enumerators (REs) using mobile phones with the Open Data Kit (ODK) software. Additional details can be found at pmadata.org [22].

RC analyses utilize cross-sectional PMA data collected in eight LMICs (ten study sites) from PMA Phase 2—Burkina Faso (national; December 2020-March 2021); Côte d'Ivoire (national; September-December 2021); Kongo Central, Democratic Republic of Congo (DRC; regional; November 2020–February 2021); Kinshasa, DRC (regional; November 2020–February 2021); Kenya (national; November–December 2020); Kano, Nigeria (regional; December 2020–February 2021); Lagos, Nigeria (regional; December 2020–February 2021); Niger (national; February–May 2022); Rajasthan, India (regional; September–December 2021), and Uganda (national; September–November 2021).

### Analytic samples

Within selected households, all females ages 15–49 years were eligible to complete the PMA Phase 2 female survey (Burkina Faso n = 6713; Côte d'Ivoire n = 4189; Kongo Central, DRC n = 2135; Kinshasa, DRC n = 2853; Kenya n = 10,008; Kano, Nigeria n = 1164; Lagos, Nigeria n = 1527; Niger n = 3867; Rajasthan, India n = 5488; Uganda n = 4875). A subset of women—those married or cohabitating and in need of contraception—were further identified for analysis (Burkina Faso n = 2767; Côte d'Ivoire n = 1561; Kongo Central, DRC n = 830; Kinshasa, DRC n = 846; Kenya n = 4588; Kano, Nigeria n = 535; Lagos, Nigeria n = 612; Niger n = 1525; Rajasthan, India n = 3017; Uganda n = 2020). Women were categorized as needing contraception if they reported that they had been sexually active within the last 12 months, were not currently pregnant, did not want to have any more children or wanted to wait at least 12 months before having more children, and identified as fecund.

### Measures

The dependent variable, past-year RC (binary), was measured via six items from the pregnancy coercion sub-scale of the RC Scale, a scale developed in the United States but previously adapted to the sub-Saharan African context. Past-year RC was assessed as an affirmative response to any of the following behaviors by a husband or partner (yes/no response to each item): (1) made you feel bad or treated you badly for wanting to use a family planning method to delay or prevent pregnancy; (2) tried to force or pressure you to become pregnant; (3) said he would leave you if you did not get pregnant; (4) told you he would have a baby with someone else if you did not get pregnant; (5) took away your family planning or kept you from going to the clinic to get family planning; (6) hurt you physically because you did not get pregnant. Item 6 was not included in the composite measure for RC but was examined as an individual item, as it was only assessed in sites where a separate gender-based violence module was implemented (Burkina Faso, Côte d'Ivoire, and Kenya) given the need for additional ethical and safety protections.

Independent variables explored as possible correlates of RC across the socioecological framework included household characteristics (residence, household wealth tertile, household composition), relationship dyad characteristics (marital status, polygynous union, age at first marriage, partner education), and individual characteristics (age, education, parity), and utilized standard assessments [19].

### Statistical analysis

All analyses were conducted per site. First, exploratory analyses examined the distribution of past-year RC and possible RC correlates. Confirmatory factor analyses assessed fit of the RC Scale using tetrachoric correlation matrixes given the binary nature of the data, with fit statistics described per Schreiber et al. criteria [23]. The prevalence of past-year RC was calculated overall and by each RC item. Post-hoc sensitivity analyses explored overlap of items to understand item precision. Bivariate logistic regression models were used to examine the association between each possible correlate and past-year RC. Correlates with p-value < 0.1 from the bivariate models were assessed for multicollinearity; if the pairwise correlation > 0.4, the most conceptually relevant variable was selected for multivariable models. All analyses were conducted in Stata 16 (College Station, TX) and were weighted to account for the complex survey design.

### Ethical protections

In line with best practices for research on sensitive topics [20, 24], REs were trained in ensuring privacy and confidentiality during the interview, with additional steps in place to enhance participant safety. Specifically, prior to asking the RC questions, the RE read the following heading: “Now I’m going to ask you a few sensitive questions about your relationship with your husband/partner. You do not have to answer these questions if you do not want to. We can pause at any time. If you do not feel comfortable answering any of the questions, let me know and I will either move onto the next statement or skip this section entirely.” At this time, the REs also re-confirmed full privacy. While violence referrals were available in the limited number of sites concurrently implementing the gender-based violence module, given limited knowledge of RC among providers in most contexts, such safeguards were not possible for the current RC research.

All respondents provided oral consent to participate. This study was approved by ethical review committees at Johns Hopkins Bloomberg School of Public Health, Comité d'Ethique pour la recherche en santé and the Ministère de la Santé et Ministère de l'Enseignement Supérieur, de la Recherche Scientifique et de l'Innovation in Burkina Faso, Comité d’Ethique de la Recherche Institut Pasteur de la Côte d’Ivoire, Comité d'Ethique de l'Ecole de Santé Publique de l'Université de Kinshasa in Democratic Republic of Congo, Kenya Medical Research Institute Ethics Review Committee in Kenya, Lagos State University Teaching Hospital Health Research Ethical Committee, Kano State Health Research Ethics Committee, Aminu Kano Teaching Hospital Research Ethics Committee in Nigeria, Comité Consultatif National d’Ethique in Niger, Indian Institute of Health Management Research University Institutional Committee for Ethics and Review of Research in India, Makerere University School of Public Health Higher Degrees, Research and Ethics Committee in Uganda.

## Results

### Sample characteristics

Across sites, most women lived in rural localities, however, in Côte d'Ivoire, residence was split evenly between urban and rural areas (Table 1). In every site but Rajasthan, the majority of women did not live with extended family. Among partnered women in Burkina Faso, Kenya, Kano, Lagos, Niger, and Rajasthan, 90% or more were married, whereas large proportions of partnered women were living with a partner outside of marriage in Côte d'Ivoire (37.0%), Kongo Central (55.8%), Kinshasa (42.7%) and Uganda (56.8%). Polygynous unions varied substantially by site—they were most common in Kano (42.0%), Burkina Faso (41.9%) and Niger (39.1%) and were least reported in Kinshasa (5.4%) and Rajasthan (0.8%). Across all sites except Kano and Niger, most women reported being 18 years or older when they first married. A secondary or higher education was most prevalent in Kinshasa (88.0%) and Lagos (84.5%) whereas no education was most common in Niger (73.2%) and Burkina Faso (69.3%). Lastly, the majority of women reported three or more live births in all sites except for Rajasthan, where just over 50% reported 1–2 births.

**Table 1:** Characteristics of married or partnered women in need of contraception across sites

	Burkina Faso (n = 2767)	Côte d'Ivoire (n = 1561)	Kongo Central, DRC^a^ (n = 830)	Kinshasa, DRC^a^ (n = 846)	Kenya (n = 4588)	Kano, Nigeria (n = 535)	Lagos, Nigeria^a^ (n = 612)	Niger (n = 1525)	Rajasthan, India (n = 3017)	Uganda (n = 2020)
Household										
Residence										
Urban	17.7	47.1	–	–	29.2	33.7	–	15.2	25.2	19.6
Rural	82.3	52.9	–	–	70.8	66.3	–	84.8	74.8	80.4
Household wealth tertile										
Lowest	38.0	40.2	32.8	36.0	36.0	29.4	31.6	31.9	26.7	34.7
Middle	32.5	30.0	34.2	34.3	34.6	33.5	35.2	34.3	35.0	34.0
Highest	29.5	29.9	33.0	29.7	29.4	37.1	33.2	33.8	38.2	31.3
Household composition										
Does not live with extended family	57.8	51.1	70.7	57.0	70.3	83.3	71.9	76.4	42.7	62.5
Lives with extended family	42.2	48.9	29.3	43.1	29.8	16.7	28.1	23.6	57.3	37.5
Relationship dyad										
Marital status										
Married	91.8	63.0	44.2	57.3	90.2	99.9	92.4	100.0	99.9	43.2
Living with partner	8.2	37.0	55.8	42.7	9.8	0.1	7.6	0.0	0.1	56.8
Polygynous union	41.9	20.8	10.6	5.4	11.1	42.0	12.2	39.1	0.8	26.4
Age at marriage										
≦ 15	4.8	12.2	5.9	4.7	8.0	37.8	1.4	33.5	13.4	11.3
> 15 and < 18	45.7	29.6	24.0	19.0	23.3	38.5	6.6	38.9	33.0	35.8
≧ 18	49.6	58.2	70.1	76.3	68.8	23.8	92.0	27.6	53.7	53.0
Partner education										
None/primary	85.1	62.7	31.8	2.9	49.8	54.3	9.4	85.8	32.0	53.4
Secondary or higher	14.9	37.3	68.2	97.1	50.2	45.7	90.6	14.2	68.0	46.6
Individual										
Age										
15–19	7.0	5.8	3.5	1.3	1.8	6.3	0.1	8.6	2.1	5.2
20–29	38.0	36.9	38.3	32.3	33.6	41.2	19.5	45.5	36.1	40.6
30–39	34.4	40.9	36.3	39.7	40.3	35.2	45.2	30.4	38.8	35.2
40–49	20.6	16.4	21.9	26.7	24.3	17.3	35.2	15.6	23.0	18.9
Education										
None	69.3	51.1	11.8	0.4	4.7	58.5	1.9	73.2	34.2	7.1
Primary	18.0	25.5	35.2	11.6	54.5	16.6	13.6	16.3	23.3	63.0
Secondary or higher	12.7	23.3	53.0	88.0	40.9	25.0	84.5	10.6	42.6	29.9
Parity										
0	1.0	1.5	1.3	1.6	1.3	0.5	1.8	2.7	4.0	0.9
1–2	29.5	33.7	30.7	33.8	33.8	18.2	34.0	23.3	52.2	27.7
3+	69.5	64.9	68.1	64.7	64.8	81.4	64.2	73.9	43.8	71.4

^a^Residence data not collected in Kongo Central, Kinshasa, and Lagos (urban samples)

### Confirmatory factor analysis

Overall, RC Scale items loaded across sites (factor loading > 0.4; Table 2). Per Schrieber et al. goodness of fit criteria, the RC Scale fit best in Uganda and Kenya based on Comparative Fit Index (CFI). In all other sites, the Comparative Fit Index (CFI) and Tucker–Lewis Index (TLI) were below recommended limits and RMSEA values over recommended limits, however, other goodness of fit metrics were suitable. Cronbach’s alpha was greater than 0.70 in all sites except Cote d'Ivoire (0.66), Kano, Nigeria (0.68), and Rajasthan, India (0.68), indicating moderate internal consistency.

**Table 2:** Factor loadings of individual reproductive coercion items and fit statistics for confirmatory factor analysis (CFA), by site, among married or partnered women in need of contraception

	Burkina Faso (n = 2767)	Côte d'Ivoire (n = 1561)	Kongo Central DRC (n = 830)	Kinshasa DRC (n = 846)	Kenya (n = 4588)	Kano Nigeria (n = 535)	Lagos Nigeria (n = 612)	Niger (n = 1525)	Rajasthan, India (n = 3017)	Uganda (n = 2020)
Factor loadings										
1. Made you feel bad or treated you badly for wanting to use a family planning method	0.77	0.84	0.81	0.77	0.85	0.94	0.85	0.75	0.78	0.85
2. Tried to force or pressure you to become pregnant	0.90	0.87	0.89	0.89	0.91	0.97	0.92	0.88	0.91	0.93
3. Said he would leave you if you did not get pregnant	0.96	0.97	0.97	0.95	0.97	0.93	0.98	0.80	0.87	0.98
4. Told you he would have a baby with someone else if you did not get pregnant	0.92	0.97	0.92	0.93	0.97	0.98	0.91	0.96	0.95	0.98
5. Taken away your family planning or kept you from going to the clinic to get family planning	0.72	0.81	0.91	0.76	0.89	0.98	0.94	0.97	0.82	0.85
Fit statistics										
CFI	0.83	0.91	0.88	0.90	0.95	0.82	0.65	0.81	0.93	0.98
TLI	0.65	0.83	0.77	0.81	0.91	0.65	0.30	0.62	0.86	0.95
RMSEA	0.43	0.33	0.38	0.31	0.25	0.62	0.84	0.48	0.26	0.18
p(RMSEA)	< 0.001	< 0.001	< 0.001	< 0.001	< 0.001	< 0.001	< 0.001	< 0.001	< 0.001	< 0.001
SRMR	0.07	0.04	0.04	0.06	0.03	0.02	0.05	0.06	0.04	0.02
AIC	27,092	13,432	7212	8223	35,799	2805	4671	14,017	29,502	15,279
BIC	27,151	13,486	7260	8270	35,863	2848	4715	14,070	29,562	15,335
CD	0.96	0.98	0.97	0.96	0.98	0.99	0.98	0.97	0.95	0.98
Alpha (α)	0.87	0.66	0.83	0.89	0.80	0.68	0.89	0.70	0.68	0.73

Desirable indices from Schreiber el al. [23]: root mean square error of approximation, RMSEA < 0.06; Comparative Fit Index, CFI > 0.95; Tucker–Lewis Index, TLI > 0.96; standardized root mean residual, SRMR < 0.08; coefficient of determination (CD). internal reliability alpha (α): ≥ 0.70

### Prevalence of past-year RC

Prevalence of past-year RC ranged from 20.3% in Kongo Central to 3.1% in Niger, with the prevalence in most remaining sites below 10% (Uganda:16.9%; Kinshasa: 11.9%; Burkina Faso: 7.1%; Kenya: 7.0%; Côte d'Ivoire: 6.2%; Kano: 5.7%; Lagos: 5.0%; Rajasthan: 3.9%; Table 3). Among women experiencing RC, they experienced one to two behaviors on average (mean range: 1.63–2.47).

**Table 3:** Prevalence of past-year reproductive coercion items, by site, among married or partnered women in need of contraception

	Burkina Faso (n = 2767)	Côte d'Ivoire (n = 1561)	Kongo Central DRC^a^ (n = 830)	Kinshasa DRC^a^ (n = 846)	Kenya (n = 4588)	Kano Nigeria^a^ (n = 535)	Lagos Nigeria^a^ (n = 612)	Niger (n = 1525)	Rajasthan, India (n = 3017)	Uganda (n = 2020)
1. Made you feel bad or treated you badly for wanting to use a family planning method	5.3	3.9	14.5	7.0	4.7	5.0	2.5	1.5	1.7	9.1
2. Tried to force or pressure you to become pregnant	2.2	3.4	11.1	5.3	3.9	2.0	2.9	0.8	1.3	10.6
3. Said he would leave you if you did not get pregnant	1.0	1.5	8.3	2.5	1.8	3.2	1.8	0.7	0.8	7.8
4. Told you he would have a baby with someone else if you did not get pregnant	1.2	1.9	8.8	2.9	1.8	3.1	2.1	0.8	1.0	8.1
5. Took away your family planning or kept you from going to the clinic to get family planning	1.4	1.0	7.4	3.3	1.8	2.1	1.1	1.0	1.8	6.1
6. Hurt you physically because you did not get pregnant^b^	0.5	0.6	ND	ND	1.2	ND	ND	ND	ND	ND
Past-year reproductive coercion experience^b^	7.1	6.2	20.3	11.9	7.0	5.7	5.0	3.1	3.9	16.9
Mean (SD), among those experiencing reproductive coercion^b^	1.62 (0.97)	1.84 (1.67)	2.13 (1.37)	1.78 (1.13)	2.01 (1.30)	2.38 (1.42)	1.90 (1.35)	1.70 (1.13)	1.67 (1.09)	2.47 (1.46)

^a^Regionally representative

^b^Item six not included in past-year estimates for comparability across all sites

Prevalence of individual RC behaviors differed by geography. The most commonly reported behavior was “made you feel bad or treated you badly for wanting to use a family planning method” in, Kongo Central (14.5%), Kinshasa (7.0%), Burkina Faso (5.3%), Kano (5.0%), Kenya (4.7%), Côte d'Ivoire (3.9%), and Niger (1.5%); however, “tried to force or pressure you to become pregnant” was most common in Uganda (10.6%) and Lagos (2.9%). Conversely, “taken away your family planning or kept you from going to the clinic to get family planning” was the most prevalent behavior in Rajasthan (1.8%), whereas this was one of the least common behaviors reported in other sites.

### Correlates of past-year RC

Bivariately, correlates of past-year RC differed substantially by setting, though there were some commonalities (Table 4). Correlates were largely concentrated at the relationship dyad level, with higher prevalence of RC reported for women living with their partners (Burkina Faso, Kinshasa, and Uganda), in polygynous partnerships (Burkina Faso, Kinshasa, Kenya, Lagos, Rajasthan, Uganda), and whose partners had lower educational attainment (Kenya, Kano, Uganda). Additionally, past-year RC was higher for women within lower household wealth tertiles (Kinshasa, Kenya, and Kano) and who were nulliparous or primiparous (Kongo Central and Kenya).

**Table 4:** Bivariate analysis of RC correlates, by site

	Burkina Faso (n = 2767)	Côte d'Ivoire (n = 1561)	Kongo Central DRC^a^ (n = 830)	Kinshasa DRC^a^ (n = 846)	Kenya (n = 4588)	Kano Nigeria (n = 535)	Lagos Nigeria^a^ (n = 612)	Niger (n = 1525)	Rajasthan, India (n = 3017)	Uganda (n = 2020)
	%*									
Total	7.1	6.2	20.3	11.9	7.0	5.7	5.0	3.1	3.9	16.9
Household										
Residence										
Urban	6.5	4.2	–	–	6.4	2.2	–	4.6	2.2	16.7
Rural	7.3	8.1^±^	–	–	7.3	7.4	–	2.9	4.5	17.0
Household wealth tertile										
Lowest	6.6	7.6	31.6	18.1	9.1	12.0	6.1	4.0	7.0	15.6
Middle	8.3	5.8	11.9^±^	11.4	5.9**	3.9**	4.8	2.4	3.8	18.9
Highest	6.5	4.8	17.9	4.9**	5.8*	2.2*	4.2	3.0	1.9^±^	16.3
Lives with extended family										
No	6.9	7.0	21.0	10.1	7.5	5.8	5.5	2.5	3.1	16.5
Yes	7.5	5.5	18.8	14.3	5.8	5.0	4.0	5.1	4.5	17.6
Relationship dyad										
Marital status										
Married	6.3	5.6	18.4	8.4	6.8	5.7	5.0	3.1	3.9	12.6
Living with partner	16.0***	7.3	21.9	16.5*	8.5	^b^	5.9	^b^	^b^	20.2***
Polygynous union	8.7*	7.1	18.3	28.4**	12.2***	4.0	10.9*	1.8^±^	32.6***	23.0**
Age at marriage										
≦ 15	5.4	9.5	19.5	20.2	6.9	6.9	^b^	2.9	6.5	17.1
> 15 and < 18	6.3	7.1	19.4	12.7	7.9	4.8	4.2	3.6	3.5	16.1
≧ 18	8.0	5.4^±^	20.7	11.7	6.8	5.5	5.3	3.3	3.1	13.6
Partner education										
None/primary	7.3	7.1	20.8	11.1	8.5	8.7	1.3	3.2	4.7	19.1
Secondary or higher	6.4	5.0	20.0	11.9	5.5**	2.1*	5.5	2.9	3.5	14.5*
Individual										
Age										
15–19	6.2	9.3	20.1	^b^	10.8	4.0	^b^	5.3	9.1	15.9
20–29	7.2	5.6	22.2	17.0^d^	7.5	6.1	3.8^d^	3.6	4.3	18.0
30–39	8.4	7.4	23.1	10.4	7.3	4.7	4.2	2.3*	3.9^±^	18.0
40–49	5.2	3.8^±^	12.5	8.5	5.5^±^	7.3	6.9	2.2^±^	2.8*	12.8
Education										
None	7.4	6.0	22.5	^b^	8.6	7.7	^b^	2.5	4.6	11.7
Primary	6.8	9.5	13.4^±^	18.7^c^	7.5	1.0^±^	4.5^c^	5.6^*^	2.7^±^	18.7^±^
Secondary or higher	6.4	3.2	24.4	11.0	6.4	4.1	5.2	3.8	4.1	14.9
Parity										
0–1	9.2	4.2	20.5	14.1	8.9	^b^	^b^	5.1	4.8	14.6
2+	6.7	6.6	14.9**	11.5	**6.7**^±^	5.1	5.4	2.8	3.7	17.3

p-value from unadjusted logistic regression, referent top category: ^±^< 0.1; *< 0.05; **< 0.01; ***< 0.001; bolding indicates p < 0.05

^a^Residence data not collected in Kongo Central, Kinshasa, and Lagos

^b^Data omitted due to small cell size

^c^In Lagos and Kinshasa, none and primary education levels were combined for bivariate logistic regression due to small cell sizes

^d^In Lagos and Kinshasa 15–19 and 20–29 age groups were combined for bivariate logistic regression due to small cell sizes

In multivariable models (Table 5), at the household level, increased household wealth was significantly associated with a decreased odds of past-year RC in Kinshasa (aOR_highest_ = 0.29; 95% CI = 0.11–0.75; p < 0.05), Kenya (aOR_middle_ = 0.68; 95% CI = 0.50–0.93; p < 0.05), and Kano (aOR_highest_ = 0.17; 95% CI = 0.03–0.98; p < 0.05). At the relationship dyad level, living with a partner while unmarried, as compared to married, was significantly associated with increased odds of past-year RC in Burkina Faso (aOR = 2.84; 95% CI = 1.70–4.75; p < 0.001) and Uganda (aOR = 1.86; 95% CI = 1.34–2.59; p < 0.001). Polygyny was the most consistent risk factor across settings, and was associated with an increased odds of past-year RC in Burkina Faso (aOR = 1.59; 95% CI = 1.00–2.27; p < 0.05), Kinshasa (aOR = 2.37; 95% CI = 1.01–5.57; p < 0.05), Kenya (aOR = 1.93; 95% CI = 1.26–2.94; p < 0.01), Lagos (aOR = 2.78; 95% CI = 1.13–6.86; p < 0.05); Rajasthan (aOR = 10.76; 95% CI = 6.10–18.96; p < 0.001), and Uganda (aOR = 1.78; 95% CI = 1.24–2.55; p < 0.01). Lastly, having a partner with a secondary or higher education level was protective against RC in Kenya (aOR = 0.67; 95% CI = 0.49–0.96; p < 0.05) and Kano, Nigeria (aOR = 0.23; 95% CI = 0.05, 0.96; p < 0.05). At the individual level, a parity of two or higher, as compared to one or fewer births, was protective against RC in Kongo Central (aOR = 0.52; 95% CI = 0.30–0.90; p < 0.05) and Kenya (aOR = 0.59; 95% CI = 0.40–0.86; p < 0.01).

**Table 5:** Multivariable logistic regression models for RC correlates, by site

	Burkina Faso (n = 2767)	Côte d'Ivoire (n = 1561)	Kongo Central DRC^a^ (n = 830)	Kinshasa DRC^a^ (n = 846)	Kenya (n = 4588)	Kano Nigeria (n = 535)	Lagos Nigeria^a^ (n = 612)	Niger (n = 1525)	Rajasthan, India (n = 3017)	Uganda (n = 2020)
	aOR (95% CI)	aOR (95% CI)	aOR (95% CI)	aOR (95% CI)	aOR (95% CI)	OR (95% CI)	OR (95% CI)	aOR (95% CI)	aOR (95% CI)	aOR (95% CI)
Total										
Household										
Residence										
Urban		Ref								
Rural		1.58 (0.62, 4.06)								
Household wealth tertile										
Lowest			Ref	Ref	Ref	Ref			Ref	
Middle			0.28 (0.07, 1.09)^±^	0.66 (0.31, 1.43)	**0.68** (0.50, 0.93)*	**0.30** (0.15, 0.60)***			0.55 (0.25, 1.24)	
Highest			0.37 (0.09, 1.57)	**0.29** (0.11, 0.75)*	0.74 (0.51, 1.08)	**0.17** (0.03, 0.98)*			0.23 (0.05, 1.04)^±^	
Relationship dyad										
Martial status										
Married	Ref			Ref						Ref
Living with partner	**2.84** (1.70, 4.75)***			1.50 (0.80, 2.81)						**1.86** (1.34, 2.59)***
Polygynous union	**1.59** (1.00, 2.27)*			**2.37** (1.01, 5.57)*	**1.93** (1.26, 2.94)**		**2.78** (1.13, 6.86)*	0.48 (0.17, 1.35)	**10.76** (6.10, 18.96)***	**1.78** (1.24, 2.55)**
Age at marriage										
≦ 15		Ref								
> 15 and < 18		0.75 (0.28, 1.98)								
≧ 18		0.60 (0.27, 1.34)								
Partner education										
None/primary					Ref	Ref				Ref
Secondary or higher					**0.67** (0.47, 0.96)*	**0.23** (0.05, 0.96)*				0.75 (0.54, 1.05)^±^
Individual										
Age										
15–19		Ref						Ref	Ref	
20–29		1.25 (0.50, 3.11)						0.77 (0.24, 2.47)	0.46 (0.14, 1.58)	
30–39		1.32 (0.48, 3.66)						0.54 (0.22, 1.33)	0.46 (0.18, 1.18)	
40–49		0.84 (0.33, 2.16)						0.59 (0.18, 1.95)	0.41 (0.10, 1.08)	
Education										
None			Ref					Ref	Ref	Ref
Primary			0.51 (0.22, 1.15)					2.05 (0.92, 4.58)^±^	0.62 (0.34, 1.14)	1.85 (0.93, 3.66)^±^
Secondary or higher			1.14 (0.55, 2.35)					1.44 (0.57, 3.60)	1.27 (0.70, 2.29)*	1.49 (0.69, 3.25)
Parity										
0–1			Ref		Ref					
2+			**0.52** (0.30, 0.90)*		**0.59** (0.40, 0.86)**					

Site-specific multivariable logistic regression, adjusted for all variables included in site column: ^±^< 0.1; *< 0.05; **< 0.01; ***< 0.001; bolding indicates p < 0.05; ^a^Residence data not collected in Kongo Central, Kinshasa, and Lagos

Variables selected based on bivariate logistic regression p-values < 0.1 and assessed for multicollinearity; most conceptually relevant variable selected if pairwise correlation > 0.4

### Post-hoc sensitivity analyses

Post-hoc sensitivity analyses aimed to understand overlap of items (data not shown); specifically, items 1 “made you feel bad or treated you badly for wanting to use a family planning method” and 2 “tried to force or pressure you to become pregnant” to understand the split from one to two items that occurred with adaptation from the original RC scale to the sub-Saharan African context; up to 5% of women in each site reported experience of one item without experience of the other. To further understand the proportion of RC cases missing in a 5-item versus 6-item measure (inclusive of physical violence with RC experience), overlap of item 6 with the other five items was examined; less than 0.01% of women in Burkina Faso, Kenya, and Cote d’Ivoire reported this RC behavior without another concurrent RC behavior.

## Discussion

These nationally or regionally representative results deem that the modified RC Scale is a reliable measure across ten settings in sub-Saharan Africa and South Asia. Moreover, these results present important nuances when examining RC by setting, indicating a need for site-specific screening and response that accounts for cultural variation in pregnancy pressures. Specifically, prevalence of past-year RC varied substantially by geography, ranging from one in five women in Kongo Central, DRC (20.3%) to 3.1% in Niger. Two countries provided estimates within multiple regions/states rather than at the national level; DRC estimates widely varied across regions (20.3% Kongo Central vs. 11.9% Kinshasa), however, were more stable across Nigerian states (5.7% Kano vs. 5.0% Lagos). Correspondingly, few correlates were consistent across sites—such risk factors were focused at the partner dyad level, including polygynous relationships and living with a partner. Accordingly, sexual and reproductive health (SRH) providers must be alert to potential partner interference and factors that may increase susceptibility to RC, while seeking to understand norms that continue to perpetuate these harmful constraints on women’s reproductive autonomy.

Foremost, these results have important measurement implications for quantifying RC in LMICs and its burden on women’s SRH. Overall, the modified RC scale showed moderate internal consistency across settings (Cronbach’s alphas range: 0.66_Cote d’Ivoire_–0.89_Kinshasa,DRC/Lagos,Nigeria_), with other fit statistics corroborating goodness of fit. Notably, some item modification has been made since the original RC Scale was published by Miller et al. [4, 17]. Specifically, one item from the original Miller RC Scale was divided into two separate items for the sub-Saharan African context based on formative work in Niger, with intent was more clearly outlined [10, 15]: “made you feel bad or treated you badly for wanting to use a family planning method” and “tried to force or pressure you to become pregnant.” Across diverse sites, the post-hoc sensitivity analyses indicated up to 5% of women exclusively reported each of these behaviors (i.e., reported item 1 but not item 2 and vice-versa). Given these distinctions, future surveys should continue to differentiate such RC behaviors.

The most prevalent item slightly differed across sites; “made you feel bad or treated you badly for wanting to use a family planning method” was most common in seven sites, whereas “tried to force or pressure you to become pregnant” was most prevalent in Lagos, Nigeria and Uganda, and “taken away your family planning or kept you from going to the clinic to get family planning” was the most common behavior in Rajasthan, India. Among women experiencing RC, one to two behaviors were experienced on average; therefore, focusing on only one of these items, similar to the DHS single-item [“Has your (husband/partner) or any other family member ever tried to force or pressure you to become pregnant when you did not want to become pregnant?”] could miss the range of RC behaviors women experience. Notably, the item most similar to the DHS single item (item 2) was not the most common across the majority of sites, suggesting that the single item currently in place in DHS may have low sensitivity and thus underestimate RC experience. Subsequent analyses are needed to understand how prevalence estimated based on this binary measure—created from the 5-item modified RC Scale—differ from the single-item measure included within the DHS once more RC data become available across contexts. Including assessment for this unique form of abuse within large-scale surveys is essential to inform prevention and response, as well as understand changes over time; future measurement refinement will seek to understand the minimal number of items needed to ensure comprehensive measurement.

This cross-site analysis emphasizes the importance of context specific factors when examining RC and pressures surrounding pregnancy, as few correlates for past-year RC were consistent across sites; the few commonalities observed across sites largely concentrated at the partner dyad level of the socioecological framework. Polygynous partnership, compared to monogamous partnership, was the most consistent correlate across sites. In Burkina Faso and Uganda, living with a partner, as opposed to marriage, significantly increased odds of past-year RC. Notably, significant differences were not seen across age groups and decreased odds for higher parity groups were only seen in Kongo Central and Kenya. While few studies have examined correlates of RC in LMICs, results are consistent with previous findings indicating that RC correlates and contributors were concentrated at the dyad level, including those related to concurrent partnerships [21]. Future research should seek to understand motivations for RC perpetration, including disentangling dyadic drivers rooted in power versus those driven by pronatalist social norms.

Some limitations exist specific to this measure for past-year RC. While the binary measure for past-year RC excludes item 6 (hurt you physically because you did not get pregnant) due to need for additional violence referrals to collect these data, less than 2% of women in the three sites that data were collected experienced this RC behavior and post hoc sensitivity analyses indicated large overlap of item 6 with other items, suggesting that these prevalence estimates are likely missing few RC cases. Notably, however, our analysis only examines pronatalist pregnancy coercion behaviors (i.e., partners seeking pregnancy and women seeking to avert pregnancy). Recent literature in sub-Saharan Africa [9, 25], however, has explored the potential for RC to act in the opposite manner, with women seeking pregnancy and men acting against their intentions to avert pregnancy (i.e. forced use of family planning). Among IPV survivors in Nairobi, an additional item was tested “forced you to use birth control;” while this item did not load in exploratory factor analyses, likely because it was measuring a unique form of RC, 13.5% of IPV survivors in urban settlements of Nairobi reported experiencing this form of RC in the past 3 months [9]. Additional research is needed to understand forced usage of family planning, rather than forced non-use, as a form of RC. Lastly, we recognize that while immense strides have been made in conceptualizing and measuring this form of abuse, this relatively narrow definition of RC excludes coercion surrounding reproductive outcomes, and specifically abortion coercion. Future work is needed to better understand the many ways that partners, and broader actors including family and community members, can counteract women’s reproductive intentions and outcomes.

Moreover, some further limitations exist specific to the present study design and analytical methods. Foremost, to understand risk of RC for women in need of contraception, this analysis excludes women who are currently pregnant. Notably, these women may be pregnant due to RC experience, leading to an underestimate of RC prevalence. Future longitudinal assessments aim to disentangle RC, contraceptive use, and pregnancy experiences to better quantify impact. All analyses are cross-sectional, limiting our understanding of temporality surrounding correlates of RC; however, the assessed correlates are largely demographic in nature and unlikely to change over the past-year period for RC assessment. Household-based data collection comprised all women ages 15–49 within the selected enumeration areas, however, in some sites (particularly Lagos and Kano, Nigeria) there were few young, nulliparous/primiparous women; as such, age and parity analyses were limited within these sites. Future research should oversample adolescents, who are at a critical stage in their life course trajectory, and may be prone to RC [25, 26]; comparison with adult populations can help disentangle possible risk associated with delayed childbearing. Similarly, duration of marriage and child sex preference should be explored at potential drivers of RC.

Local policymakers are well positioned to institute culturally appropriate programs to mitigate RC and its impact. Notably, some factors were found to be protective against RC, and further research should seek to understand their role in preventing RC perpetration. Specifically, in Kinshasa, DRC and Kano, Nigeria, RC experience declined with increasing household wealth tertile, and in Kenya and Kano partner education of secondary school or higher was protective against past-year RC; in Kano, odds of RC reduced by nearly 80% for those with a more educated partner. While continuing to institute programs to expand access to education and empower women and girls, these results speak to the need to simultaneously educate men, both generally and specifically to dismantle patriarchal norms that promote men as the ultimate decision-makers in household matters, including contraceptive use. Moreover, in high RC prevalence contexts, such as DRC and Uganda, screening for RC must be institutionalized within all SRH services, and specifically family planning services; protection against unintended pregnancy is imperative when nearly one in five women are experiencing this form of reproductive violence. Interventions such as Addressing Reproductive Coercion within Healthcare Settings (ARCHES) [27, 28] can provide a framework for training family planning providers on how to recognize, screen, and prevent recurrent RC; this intervention has been implemented in the United States, Kenya, and Bangladesh and may prove useful to other contexts if culturally adapted. Though RC prevalence and correlates vary across contexts, this form of abuse is ultimately rooted in power and control—counteracting harmful norms will require both empowering women and girls in their reproductive choices and bolstering men as supportive partners in reproductive decision-making.

## Data Availability

Data is available upon request from www.pmadata.org.

## Abbreviations

ARCHES: Addressing Reproductive Coercion in Health Settings
CFA: Confirmatory factor analysis
CFI: Comparative fit index
DHS: Demographic and Health Survey
DRC: Democratic Republic of Congo
IPV: Intimate partner violence
LMIC: Low- and middle-income country
ODK: Open Data Kit
PMA: Performance Monitoring for Action
RC: Reproductive coercion
RE: Resident enumerator
RMSEA: Root mean square error of approximation
SRH: Sexual and reproductive health
TLI: Tucker Lewis Index
