# Correction: Prevalence and correlates of reproductive coercion across ten sites: commonalities and divergence

**DOI:** 10.1186/s12978-023-01591-2

**Published:** 2023-03-30

**Authors:** Shannon N. Wood, Haley L. Thomas, Georges Guiella, Fiacre Bazie, Rosine Mosso, Raimi Fassassi, Pierre Z. Akilimali, Mary Thiongo, Peter Gichangi, Sani Oumarou, Funmilola M. OlaOlorun, Elizabeth Omoluabi, Anoop Khanna, Simon Peter Sebina Kibira, Fredrick Makumbi, Michele R. Decker

**Affiliations:** 1 Department of Population, Family and Reproductive Health, Johns Hopkins Bloomberg School of Public Health, 615 N Wolfe St, E4009, Baltimore, MD 21205 USA; 2 Department of Population, Family and Reproductive Health, Bill & Melinda Gates Institute for Population and Reproductive Health, Johns Hopkins Bloomberg School of Public Health, Baltimore, USA; 3 Institut Superieur des Sciences de la Population (ISSP/University of Ouagadougou), Ouagadougou, Burkina Faso; 4 Ecole Nationale Superieure de Statistique et Appliquee d’Abidjan (ENSEA), Abidjan, Côte d’Ivoire; 5 Kinshasa School of Public Health, Kinshasa, Democratic Republic of Congo; 6 International Centre for Reproductive Health-Kenya, Nairobi, Kenya; 7 Technical University of Mombasa, Mombasa, Kenya; 8 Department of Public Health and Primary Care, Faculty of Medicine and Health Sciences, Ghent University, Ghent, Belgium; 9 Institut National de la Statistique du Niger, Niamey, Niger; 10 College of Medicine, University of Ibadan, Ibadan, Nigeria; 11 University of the Western Cape, Cape Town, South Africa; 12 Indian Institute of Health Management Research, Sanganer, Jaipur, India; 13 Makerere University School of Public Health, Kampala, Uganda; 14 Johns Hopkins School of Nursing, Baltimore, USA

**Correction: Reproductive Health (2023) 20:22** 10.1186/s12978-023-01568-1

After publication of this article [1], the authors reported that in the ‘Ethics approval and consent to participate’-section, one of the ethical review committees was incorrectly given as ‘Kenya Medical Research Institute Ethics Review Committee’, but should have been ‘Kenyatta National Hospital/University of Nairobi Ethics & Research Committee’.

The original article [1] has been corrected.
